# 815 Inhalation Injury Is Associated with a Unique Cytokine Profile in Burn Patients

**DOI:** 10.1093/jbcr/iraf019.346

**Published:** 2025-04-01

**Authors:** Dhanushka Vitharana, Sophia Trinh, Paige Deville, Abdul-Razak Masoud, Ada Ozcan, Kaitlyn Andre, Jeffrey Carter, Herbert Phelan, Jonathan Schoen, Victoria Miles, Alison Smith

**Affiliations:** Louisiana State University Health Sciences Center - New Orleans; Louisiana State University Health Sciences Center - New Orleans; Louisiana State University Health Sciences Center - New Orleans; Louisiana State University Health Sciences Center - New Orleans; Louisiana State University Health Sciences Center - New Orleans; Louisiana State University Health Sciences Center - New Orleans; Louisiana State University Burn Center; University Medical Center, New Orleans Burn Unit; Louisiana State University Health Sciences Center - New Orleans; University Medical Center New Orleans; Louisiana State University Health Sciences Center - New Orleans

## Abstract

**Introduction:**

Inhalation injuries from burns are associated with worse patient outcomes including increased risk of pneumonia and progression to multisystem organ failure. Increased concentrations of several pro- and anti-inflammatory cytokines have been found in non-survivors of inhalation injuries compared to survivors. The aim of this study was to examine how the inflammatory profile of burn patients with inhalation injuries differs from those without inhalation injuries. It is hypothesized that patients with inhalation injuries will demonstrate a different cytokine profile to burn patients without inhalation injuries.

**Methods:**

After obtaining IRB approval, adipose tissue was collected from adult burn patients presenting to an ABA-Verified Burn Center from 2022-2024 during initial excision. Thermal, electrical and chemical burns were included, as well as burn patients with concomitant trauma. Adipose-derived stem cells (ADSCs) were extracted and the supernatant was collected. Cytokine analysis was performed using a 10-analyte multiplex assay. The cytokines studied were IFN-γ, IL-1β, IL-4, IL-6, IL-10, IL-13, IL-17A, TGF-α, TNF-α, FGF-2, MCP-1 and VEGF. Patients were stratified based on the presence of inhalation injury. Statistical analysis was performed using the Mann-Whitney U test.

**Results:**

Of the 28 patients studied, 15 patients (54%) sustained inhalation injuries. No significant differences in age, body mass index, sex, % total body surface area burn, blood alcohol level on admission, smoking status or mortality were observed between groups (p>0.05). In patients with inhalation injuries, IL-8 was significantly decreased as compared to patients without inhalation injury (p=0.03). No significant differences in the other cytokines analyzed were identified between groups (p>0.05).

**Conclusions:**

This study suggests that adipose derived stem cells from burn patients with inhalation injury produce decreased levels of IL-8 in adipose tissue. IL-8 is a pro-inflammatory cytokine that activates neutrophils and is involved in angiogenesis and cell growth. Elevated plasma IL-8 is implicated in development of sepsis following burn injuries and positively correlates with inhalation injury severity. It is possible that IL-8 produces a different effect in adipose tissue compared to plasma. Ultimately, investigating the cytokine profile of adipose tissue following concomitant inhalation and burn injury may further elucidate the role the local inflammatory response plays in the systemic inflammatory response seen in major burn injury.

**Applicability of Research to Practice:**

Studying the cytokine profile of ADSCs may help broaden our understanding of the often detrimental systemic inflammatory response produced in response to major burn injury.

**Funding for the Study:**

AAST Trauma Research Scholarship

